# Recent Advances in Recognition Receptors for Electrochemical Biosensing of Mycotoxins—A Review

**DOI:** 10.3390/bios13030391

**Published:** 2023-03-17

**Authors:** Manpreet Kaur, Jyoti Gaba, Komal Singh, Yashika Bhatia, Anoop Singh, Narinder Singh

**Affiliations:** 1 Department of Chemistry, Punjab Agricultural University, Ludhiana 141004, India; 2 Department of Chemistry, Indian Institute of Technology, Ropar 140001, India

**Keywords:** biosensors, mycotoxins, recognition receptors, electrochemical biosensing

## Abstract

Mycotoxins are naturally occurring toxic secondary metabolites produced by fungi in cereals and foodstuffs during the stages of cultivation and storage. Electrochemical biosensing has emerged as a rapid, efficient, and economical approach for the detection and quantification of mycotoxins in different sample media. An electrochemical biosensor consists of two main units, a recognition receptor and a signal transducer. Natural or artificial antibodies, aptamers, molecularly imprinted polymers (MIP), peptides, and DNAzymes have been extensively employed as selective recognition receptors for the electrochemical biosensing of mycotoxins. This article affords a detailed discussion of the recent advances and future prospects of various types of recognition receptors exploited in the electrochemical biosensing of mycotoxins.

## 1. Introduction

Chemical and electrochemical sensors are an important field of scientific research for their practical applications [1,2,3]. An electrochemical chemical sensor transforms the information on the nature and concentration of the analyte into an electrical signal [4]. Various sensors have been reported for the sensing of gases, water pollutants, and mycotoxins in food for safer health and working environment. Mycotoxins are a group of toxic chemicals naturally produced by various species of fungi and molds. They have been detected in a variety of foods and crops. Because of their chemical stability, they can withstand high temperatures of cooking and food processing. Dairy goods, alcoholic beverages, and agricultural commodities are the main sources of mycotoxins [5]. Mycotoxins have severe negative health impacts causing increased emergence of gastrointestinal, hepatic, and carcinogenic diseases [6]. There are around four hundred commonly occurring mycotoxins that have been reported, although only a handful of them are harmful to human health [7]. A collaboratively convened international committee, i.e., Joint Expert Committee on Food Additives (JECFA), by World Health Organization (WHO) and Food and Agricultural Organization (FAO), is responsible for evaluating risks, setting the codes of practice, exposure, and tolerable daily intake limits of mycotoxins in various food products [8]. Most common examples of mycotoxins include aflatoxins (AFs), ochratoxins, patulin, trichothecenes, zearalenone (ZEA), T-2 toxin, nivalenol, HT-2 toxin, citrinin, ergot alkaloids, Penicillin Roquefort (PR) toxin, cyclopiazonic acid, sterigmatocystin, etc. [5].

**Figure 1:**
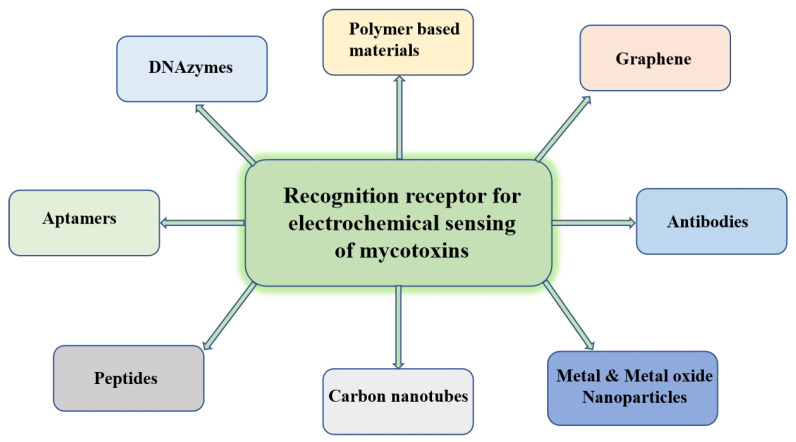
Recognition receptors for electrochemical sensing of mycotoxins. Mechanism of electrochemical biosensing.

There are advanced analytical methods, such as high-performance liquid chromatography–mass spectrometry (HPLC–MS), gas chromatography (GC), etc. which are widely used to monitor mycotoxins. However, these methods for sensing require a great level of sophistication and control. Electrochemical sensing is an alternate methodology with ease of use, fast response, and high sensitivity. During the process of electrochemical sensing, the electrode acts as the core, and at the surface of the electrode, the recognition element is present. As the analyte and recognition element combine, the reaction signal is transformed into an electrochemical signal. A major demand in this field is the designing of recognition receptors with high sensitivity, selectivity, and stability. In the last decade, the scientific fraternity has been working on the designing of electrochemical biosensors for the recognition of mycotoxins. Till now, a number of reviews on the presence of mycotoxins in various food and feed products, their health impacts, and their biological degradation are available [9,10,11,12]. A few reviews on current methods of detection of mycotoxins are also available. However, the present review is focused on detailed insights into the recognition receptors for the electrochemical biosensing of mycotoxins. In this review article, different recognition receptors for mycotoxins viz polymer-based materials, DNAzymes, antibodies, peptides, aptamers, graphene, carbon nanotubes, and metal and metal oxide nanoparticles are discussed in detail (Figure 1). Their mode of action has been elaborated. The present status of research in this field has been discussed, and the future potential has been highlighted.

An electrochemical biosensor consists of two main units, recognition receptor and signal transducer. Natural/artificial antibodies, aptamers, molecularly imprinted polymers (MIP), peptides, and DNAzymes have been extensively employed as selective recognition receptors for electrochemical biosensing [13]. The generation of the signal is based on redox reactions, which are shown by redox-active chemical species, viz. enzymes, polymers, catalysts, nanoparticles, quantum dots, carbon nanotubes, etc., attached covalently to the aptamers, for example [14]. In addition to the recognition receptor, a biosensor also comprises a transduction unit that modifies the signal produced by the binding/reaction of the analyte with the recognition receptor into an amplified electric signal. The change in voltage or current is produced as a result of the interaction of the target with the recognition element. The oxidation-reduction reactions (involving the gain or loss of electrons) occur on the surface of the electrode. These are monitored using different electrochemical methods. Electroanalytical methods can be classified into three major types: voltammetry, electrochemical impedance spectroscopy (EIS), and chronoamperometry, on the basis of differences in input and observable signals. Voltammetry measures how varying electrode current with the applied voltage during an electrochemical reaction and can be further classified into linear-sweep voltammetry (LSV), cyclic voltammetry (CV), differential-pulse voltammetry (DPV), and square-wave voltammetry (SWV) [15,16]. LSV is employed frequently for qualitative analysis only where a one-way linearly varying voltage (potential sweep) is applied between the working and counter electrode followed by current measurement. In the case of CV, a voltage cycle (multiple potential sweep) is applied, and then the resulting current is measured for qualitative as well as quantitative determination of the analyte. The sensitivity of CV can be further improved with DPV, where linear voltage input is substituted with a pulsed form for the enhancement of peak resolution and detection of trace levels of analytes. SWV, on the other hand, involves the application of a potential waveform having two pulses in opposite directions (staircase potential and alternating square wave) to the stationary electrode and measurement of current at the end of both forward and reverse pulse. Due to the low detection limits and high sensitivity, the SWV technique has emerged as a promising electroanalytical method for the simultaneous biosensing of multiple analytes. EIS is altogether different from voltammetry, where instead of current or voltage, an alternating potential wave having a small amplitude and varying frequency is applied to the electrode, and the impedance with other electrochemical properties is measured as a response signal. The voltammetric methods, such as CV, DPV, LSV, and SWV, are better categorized as electroanalytical, and EIS is considered an electrochemical characterization technique. Chronoamperometry is a faster method that involves the input signal as a step (single or double) voltage followed by measurement of electrode current as a function of time. This method can also be used to assess the stability and repeatability of electrochemical biosensors by monitoring the variations in the current curve obtained after feeding single/double step potential. In conclusion, all these methods can be used for the electrochemical biosensing of analytes, and each method has its own advantages. LSV method provides analysis with a very small relative standard deviation but also with low recovery as compared to DPV. SWV can provide an excellent biosensing platform due to its high sensitivity as compared to the other methods. Table 1 summarizes the different electroanalytical methods employed by the researchers for the electrochemical sensing of various mycotoxins. For AFB1 detection, the most sensitive method was based on SWV, which could detect this mycotoxin with a LOD of 0.6 × 10^−7^ ng/mL in corn samples (Table 1). Similarly, EIS, CV, and DPV were observed as the most sensitive electroanalytical techniques for the detection of OTA (Table 1).

In the next section, different recognition elements have been discussed.

## 2. Recognition Receptors

### 2.1. Aptamers as Recognition Receptors

The word “aptamer” originated from the Latin word “aptus”, which means “to fit” [17]. Aptamers comprise 15–90 single-stranded synthetic nucleic acid chains, which might get folded into three dimensions and bind to a target varying from huge proteins to tiny molecules, such as amino acids or drugs, with extreme specificity and affinity [18]. Nowadays, aptamers have been extensively utilized for pathogen recognition [19], clinical diagnostics of cancer and stem cells [20], environmental protection for detecting pollutants [21], as well as in food safety for the recognition of mycotoxins [22]. For biosensing of various analytes, these single-stranded oligonucleotide molecules acquire special 3-D conformations, such as hairpins, stems, loops, pseudoknots, bulges, G-quadruplexes, or triplexes [23]. These conformations are caused by adaptive folding due to molecular forces, such as van der Waals, electrostatic interactions, hydrogen bonding, and π-stacking. 

Aptamers can be constructed using the computational method “Systematic Evolution of Ligands by Exponential Enrichment (SELEX)”, following steps of adsorption, recovery, and amplification [24]. Aptamers have proven to be cheap, easy to prepare, and exhibit higher stability. Since the invention of aptamers, beyond 2000 types of aptamers have been developed using the SELEX approach [17]. Synthetically prepared aptamers can be simply altered to extend their lifetime in the bloodstream, target them to specific locations, or permit their immobilization. These can be specially customized to incorporate the labels and functional groups needed for their immobilization on the electrode’s surface during the electrochemical detection of mycotoxins, antibiotics, drugs, heavy metals, and ions [25]. Aptamers have become the most preferred alternatives for antibodies in biosensor development because of their potential to selectively bind certain analytes via non-covalent interactions and their reversible reactions [26]. Neither modification nor immobilization of aptamers generally results in loss of function, whereas antibodies do. Aptamers have several benefits against antibodies, for instance, thermal equilibrium, economical, easy chemical change, reusability, and stability toward hydrolysis [27,28]. With their characteristics in specificity, easy screening, and high stability, aptamers have become prominent recognition elements for biosensor platforms.

#### 2.1.1. Immobilization

**Figure 2:**
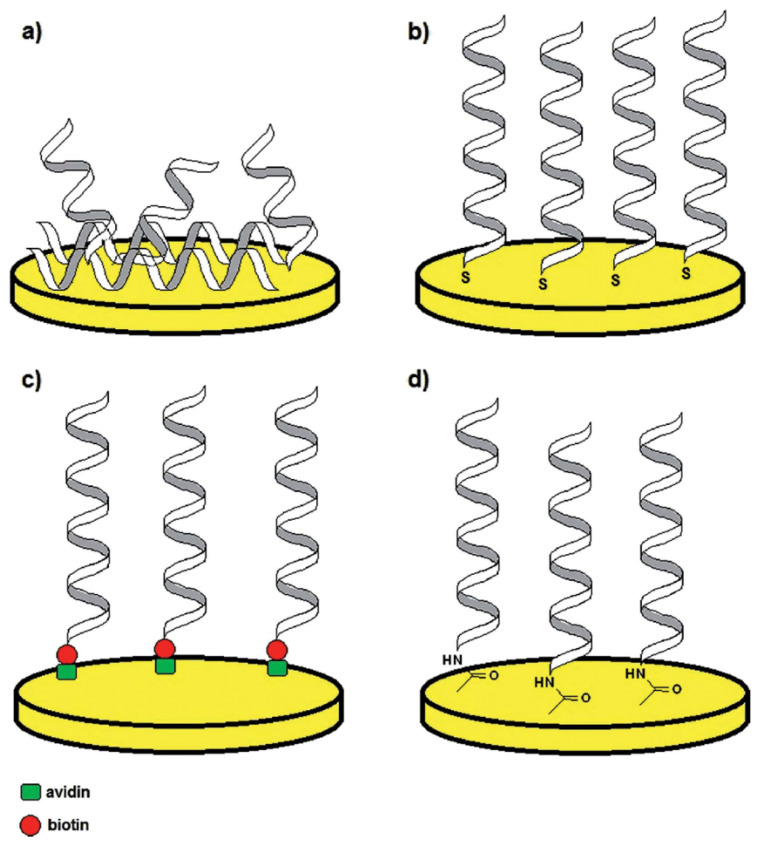
Examples of aptamer immobilization methods: via physical adsorption (**a**); thiol self-assembled monolayers (SAMs) (**b**); avidin-biotin complex formation (**c**); and surface activation with EDC/NHS (1-ethyl-3-(-3-dimethylaminopropyl) carbodiimide hydrochloride/N-hydroxysuccinimide) (**d**). Reproduced from [29] with permission from the Royal Society of Chemistry.

The primary initiative in preparing an aptamer-based biosensor is to immobilize a suitable aptamer on the conducting surface, such as a carbon nanotube, metal, or polymer electrode. Aptamers can be deposited straight on the electrode surface or indirectly through chemical coupling. The selection of an immobilization approach relies on several components, such as the properties of the target analyte, the type of biological recognition element, the transducer surface, and the operating parameters of the aptasensors [29]. To use aptamers as molecular identification agents in biosensors, it is essential to develop methods for aptamer immobilization so that they can maintain their biophysical properties and association capacities [30]. With the advantage of their compact size and adaptability to enable effective immobilization in high-density monolayers, which is essential in miniaturized systems, such as biosensors, these bioreceptors are mounted on the electrode in a stable and consistent way. In recent years, numerous immobilization strategies have been developed, including electrodeposition, chemisorption, physisorption, and (strept) avidin-biotin interactions [29]. Figure 2 shows four different immobilization methodologies. As per the driving forces of the process, immobilization could be classified into three groups, i.e., covalent, physical, and affinity immobilization [31].

**Figure 3:**
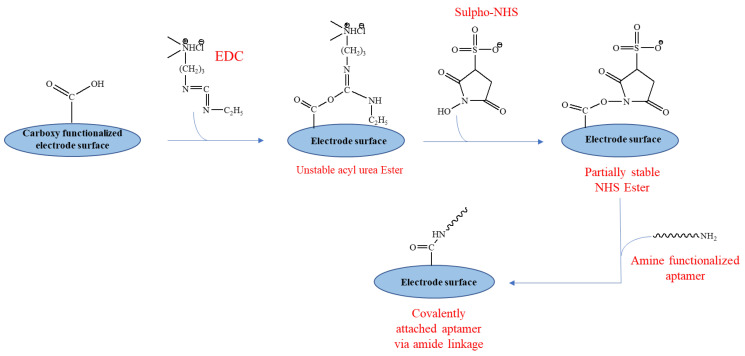
Schematic representation of EDC/NHS chemistry.

Aptamer immobilization via direct attachment to the gold surface involves modification of aptamer via thiol group (-SH), sulfides (R–S–R), and disulfides (R–S–S–R) which exhibit significant assimilation onto metal surfaces, such as silver, gold, copper or platinum. These sulfur clusters adsorb impulsively onto the metal surface, generating a stable mono-molecular layer known as the self-assembled monolayer (SAM). Further, the reinforcement of immobilized aptamers with mercaptohexanol (MCH) helps in displacing the non-specifically adsorbed portions of the aptamers and confirms their upright alignment [32]. The covalent binding of the aptamer is performed via the formation of resonance-stabilized amide linkage with the electrode surface. In this process, functional groups, such as−NH_2_, −OH, and −COOH, are incorporated onto the surface of an electrode. This functional group interacts with a customized aptamer that has a corresponding functional group. The most frequently used approach is a carbodiimide-mediated process that employs the reagents EDC and NHS for linking the aptamer to the surface of the electrode via an amide bond (Figure 3) [33]. In comparison to the conventional EDC/NHS coupling, an amine coupling mechanism using cysteamine/glutaraldehyde is extensively used. Initially, cysteamine forms a SAM by binding to the electrode through its thiol groups, onto which glutaraldehyde attaches to generate aldehyde groups for the subsequent binding of aminated molecules [34]. By using diazonium salts, covalent surface modifications of graphene-based materials are easily accomplished as they permit the addition of several chemical groups, such as−B(OH)_2_, −COOH, and −C≡CH. Following the release of N_2_ molecules and the formation of radicals, diazonium salts form covalent bonds with the sp^2^ hybridized carbon lattice atoms of graphene oxide films [35]. Aptamers can also be immobilized using non-covalent interactions between avidin (or its derivatives) and biotin. In this method, a biotinylated aptamer can be immobilized on the surface coated with avidin or its derivatives (neutravidin and streptavidin) via noncovalent interactions [32].

#### 2.1.2. Classification

The electrochemical aptasensors have been categorized into three groups based upon the change induced by the analyte, such as conductivity change (the analyte bonding “turns on” the conductivity of the surface-bound DNA-aptamer structures), the conformational change (analyte interaction produces a change in the arrangement of the surface-immobilized aptamer strands), and configurational change (analyte interaction leads to either an association or dissociation of the sensor developed) [36].

Other classifications of aptasensors include label-free, labeled, signal-off (with decreased activity), and signal-on (with improved activity). The label can be covalently bonded to the aptamer, and the aptasensor’s behavior to the target relies on the response of the label following the production of an aptamer-target complex. Until now, a variety of substances, including enzymes (glucose oxidase (GOD), alkaline phosphatase (AP), and horseradish peroxidase (HRP)) and nanomaterials (carbon-based nanomaterials, metal-based nanomaterials, quantum dots (QDs), and polymers) have been used as labels [37]. The sandwich assay scheme is used to monitor various targets where one aptamer is immobilized to the sensor surface, whereas a secondary labeled aptamer/protein/antibody is employed for signal amplification. The target with two distinct binding sites is trapped between them [38]. In the label-free type of aptasensors, the binding of the target and immobilized aptamer can be detected using redox mediators (methylene blue and ferricyanide/ferrocyanide redox couple), which disperse on the surface of the electrode and show electrochemical oxidation/reduction via heterogeneous electron transfer to or from the electrode [39]. 

#### 2.1.3. Advances in Aptamer Research

Since 2008, a series of studies have been reported involving aptamers specific for mycotoxins using electrochemical [40], colorimetric [41], and fluorescent transducers [42]. Among them, electrochemical aptasensors have gained interest recently due to their simplicity, affordability, sensitivity, and ease of miniaturization, which is widely useful in their on-site determination [43]. Classical technologies have been utilized for the electrochemical aptasensing of ochratoxin A (OTA) [40,44], fumonisin B1 (FB1) [45], aflatoxin B1 (AFB1) [41,46], zearalenone [18], or aflatoxin M1 (AFM1) [47].

Azri et al. [18] prepared a label-free competitive electrochemical aptasensor via a covalently modifying gold electrode (AuE) with zearalenone employing 1,4-phenylene diisocyanate and cysteamine-hydrochloride as linkers for analyzing its concentrations in maize samples using SWV approach. For a given quantity of aptamer in each sample, the free zearalenone in the solution competed with the immobilized zearalenone on the AuE. It was observed that TAT, TAC, and CAT were often found in the sequences and could be considered essential components of potential binding sites. The quantity of aptamer that could bind to the fixed zearalenone on the surface of AuE was restricted by enhancing the free zearalenone concentration in the solution, which led to a high current response. 

**Figure 4:**
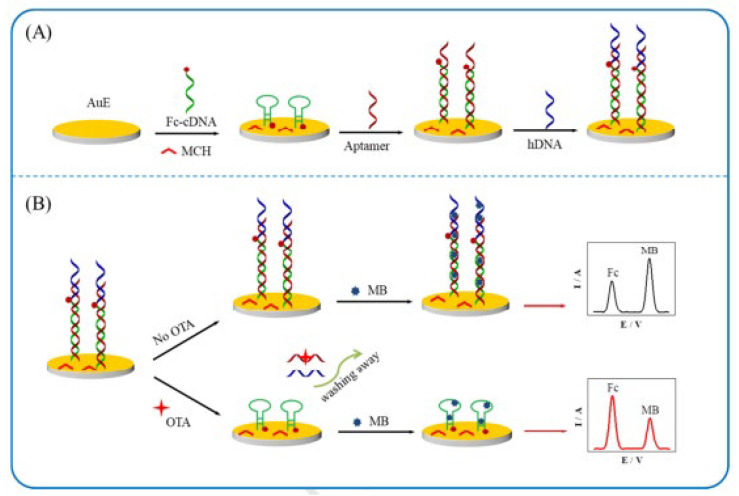
Schematic representation of the fabrication and detection process of the aptasensor. (**A**) design of aptasensor by stepwise addition of Fc-cDNA, OTA aptamer and hDNA on Au electrode (**B**) special affinity for OTA caused dissociation of aptamer and formation of hairpin structure. Reproduced from [40]. Copyright 2020 Elsevier. hDNA: helper DNA, dsDNA: double-stranded DNA, cDNA: complementary DNA, MB: methylene blue; Fc-DNA: Ferrocene-labeled complementary DNA.

For the sensing of OTA, Zhu et al. [40] devised a ratiometric electrochemical aptasensor depending on the interaction of methylene blue and DNA using a dual signal amplification approach (Figure 4). The aptasensor was comprised a three-electrode system, i.e., gold, silver/silver chloride (potassium chloride), and a platinum wire as working, reference, and counter electrode, respectively. The aptasensor was designed by the gradual addition of ferrocene-labeled complementary DNA, the OTA aptamer, and hDNA to devise dsDNA structures onto the gold electrode (Figure 4A). The cDNA had complementary constituents (TGTCCG, CGGACA) in its sequences that potentially formed a hairpin structure. When OTA was present, its strong attraction to the aptamer caused the hDNA and aptamer to separate from the electrode surface and led to the development of a cDNA hairpin-like structure. Consequently, a small oxidation current of ferrocene (I_Fc_) went up since Fc was closer to the electrode, and a large oxidation current of methylene blue (I_MB_) went down due to the poor binding ability of cDNA to methylene blue. Thus, I_Fc_/I_MB_ ratio was used to measure OTA concentrations (Figure 4B). The aptasensor thus obtained signified its enhanced analytical capabilities and extensive potential administration for the detection of mycotoxins.

**Figure 5:**
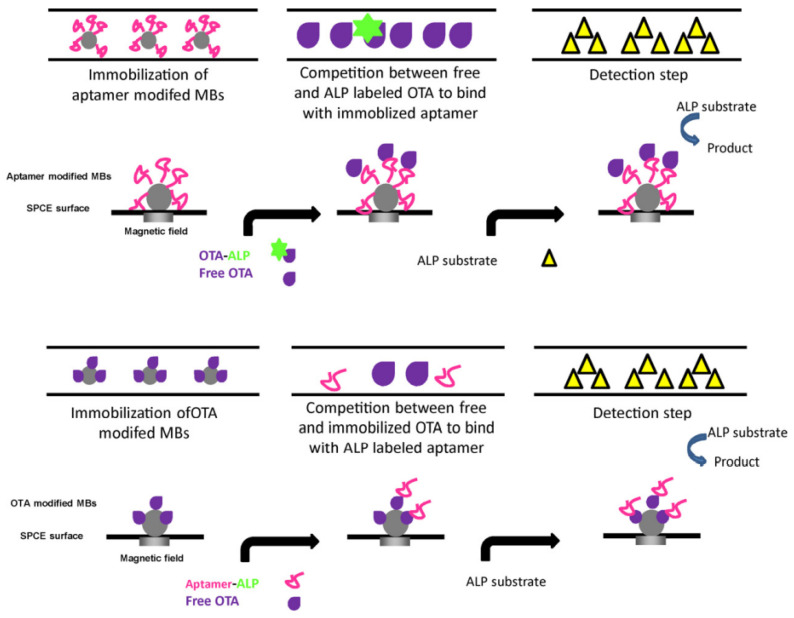
Schematic representation of e-principle for the automated flow-based electrochemical aptasensor using direct and indirect competitive formats. Reproduced from [44]. Copyright 2013 Elsevier.

Using direct and indirect competitive methods, an automatic flow-based electrochemical aptasensor (5′-GAT-CGG-GTG-TGG-GTG-GCG-TAA-AGG-GAGCAT-CGG-ACA-3′) was prepared for the on-line detection of OTA with modified magnetic beads and an aptamer [44]. The designed aptasensor was demonstrated using direct and indirect competitive approaches in conditions of continuous/stopped flow (Figure 5). In the direct assay, biotin-labeled and free OTA contended in for binding with immobilized aptamer on screen-printed carbon electrode (SPCE) surface, and further, electrochemical detection was achieved via alkaline phosphatase avidin (avidin–ALP) couple. In the indirect approach, free OTA and immobilized OTA competed for aptamer in the solution phase, and then, Avidin–ALP couple was employed to carry out electrochemical sensing. The introduction of the aptamer into the flow device has resulted in an enhancement in the sensitivity to detect OTA at trace levels. The designed flow-based aptasensor could be used by unskilled persons for the on-the-spot detection of OTA in medical, environmental, and food analysis due to its simplicity and automation.

Suea-Ngam et al. [48] fabricated an electroanalytical aptasensor (5′-HS(CH_2_)_6_ AAA AAAAAA AGAT CGG GTG TGG GTG GCG TAA AGG GAG CAT CGG ACA-3′) based on a DNA aptamer, exonuclease I (for digesting unbound aptamers) and silver metallization (for signal amplification) for detection of OTA. Exo. I eliminated free aptamers by digesting single-stranded DNA, but it was found to be ineffective against the G quadruplex generated upon OTA binding. The signal was significantly improved by adding AgCl and then using a double reduction technique to metalize the target-bound aptamers. The result obtained demonstrated exceptional biosensing capabilities, including high selectivity, high sensitivity, better stability, and excellent reproducibility possessed by the electrochemical aptasensors. The designed aptasensor was then used for the determination of OTA in beer, with a recovery rate of 96.6% to 109.7% and less than a 5% standard deviation.

On the principle of exonuclease-catalyzed target reprocessing, a novel “signal-on” aptamer (DNA 1: 5′ -Fc–CCG ATG CTC CCT TTA CGC CAC CCA CAC CCG ATC GG-(CH_2_)6-SH-3′; DNA 2: 5′-AAA GAT CGG GTG TGG GTG GCG TAA AGG GAG CAT CGG ACA-3′)-based sensor for detection and quantification of wheat samples was prepared by Tong et al. [49]. OTA formed a complex with aptamer, which led to the separation of aptamer from double-stranded DNA and the formation of the hairpin-like structure of probe DNA. Exonuclease, which selectively digested the aptamer, was used to release the OTA from the aptamer–OTA couple for analyte regeneration. This innovative method claimed the benefits of being simple, economical, and sensitive for the evaluation of OTA in food samples.

**Figure 6:**
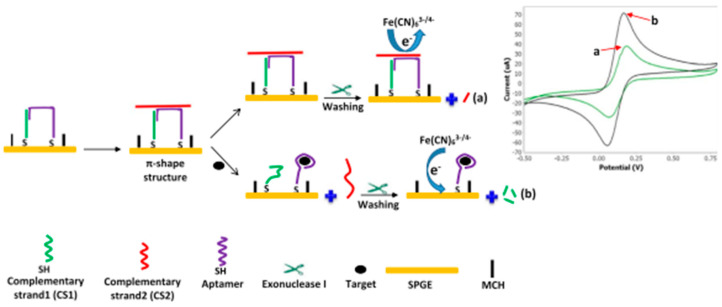
Fabrication process of the developed π-shape electrochemical aptasensor for AFB1. In the absence of AFB1, most parts of the π-shape structure were intact, and redox mediator did not have access to the surface of electrode, leading to a weak current signal (**a**). In the presence of AFB1, Apt bound to AFB1 and left the CSs. Exo I digested CS1, resulting in more access of [Fe (CN)_6_] ^3−/4−^ to the surface of electrode and generated a strong current signal (**b**). Reproduced from [50]. Copyright 2017 Elsevier.

A novel electrochemical aptamer-based sensor having aptamer-complementary strands of aptamer (CSs) conjugate and exonuclease I (Exo I) was reported by Abnous et al. [50] (Figure 6). The electrochemical biosensor used the π-shaped structure of the Aptamer–CSs couple as a double-layered physical barricade to prevent the redox mediator [Fe (CN)_6_]^3−/4−^ from entering the AuE and Exo I-aided signal amplification. Without incorporating AFB1, the π-shaped structure on AuE and major sequences remained unaffected by Exo I. Therefore, [Fe (CN)_6_]^3−/4−^ had inadequate access to the electrode surface, resulting in a poor electrochemical signal (Figure 6a). When AFB1 was present, aptamer interacted with it and was removed from the complimentary strands (CS1 and CS2). The π-shaped structure was, therefore, disassembled (Figure 6b). The inclusion of Exo I to the electrode surface aided in the digestion of CS1 on the electrode surface, and thus, [Fe (CN)_6_]^3−/4−^ gained greater contact with the electrode surface, which further resulted in strong current signals. The applicability of the designed aptasensor was also verified by measuring AFB1 levels in human blood and grape juice samples.

**Figure 7:**
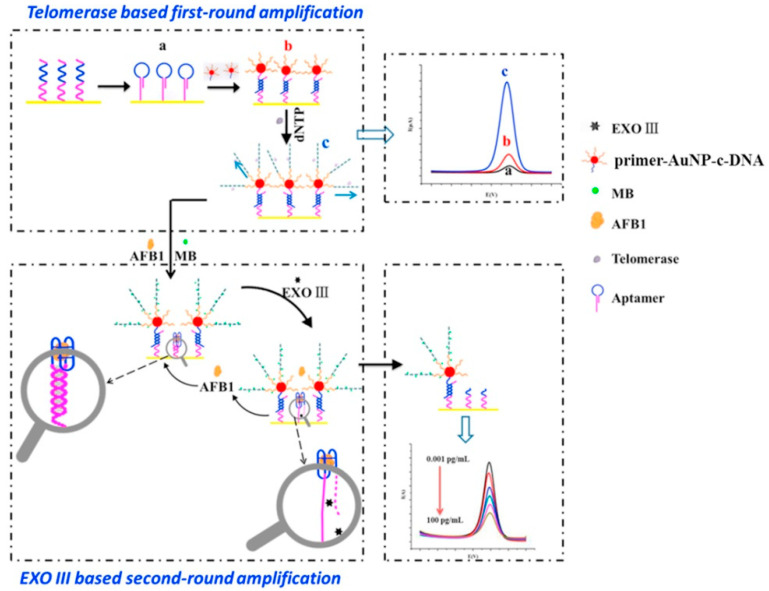
The schematic process of signal-amplified electrochemical aptasensor for AFB1 detection ((**a**) formation of stem-loop structure of the immobilized probe; (**b**) immobilization of TS-primer-AuNP-cDNA onto the sensing interface; and (**c**) telomerase-based first-round amplification). Reproduced from [51]. Copyright 2016 Elsevier.

A “signal-off” electrochemical aptasensor for trace level determination of AFB1 was designed by Zheng et al. [51] using an aptamer as the recognition unit and telomerase and exonuclease III as signal enhancement units (double signal amplification) at pH 7.4 and temperature of 298K. In order to increase the signal response span of the aptasensor, single-stranded DNA probes were amplified using telomerase and subsequently attached to the surface of gold nanoparticles (AuNPs). Following the recognition of target AFB1, EXO-III-based amplification was employed, which hydrolyzed the 3′-end of the double-stranded DNA and released the bound AFB1 so that it could rejoin the sensing system and participate in the subsequent recognition-sensing cycle (Figure 7). 

**Figure 8:**
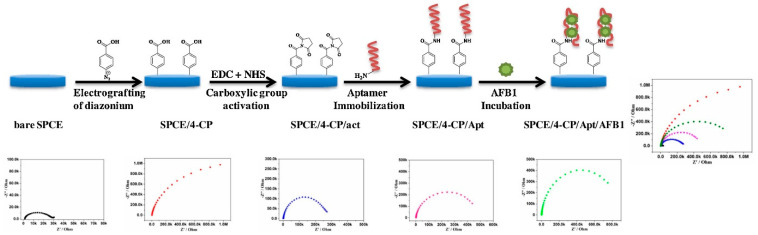
Schematic representation of the electrode fabrication. Reproduced from [22]. Copyright 2017 Elsevier.

Goud et al. [22] designed and prepared a disposable and portable label-free electrochemical aptasensor (5′-TGGGGTTTTGGTGGCGGGTGGTGTACGGGCGAGGG-3′) for the efficient detection of AFB1 in alcoholic beverages. The detection of AFB1 was based on specific identification by the compact aptamer monolayer bound covalently on SPCE via diazonium coupling and EDC/NHS reaction chemistry, followed by characterization with CV and EIS (Figure 8). During the whole analytical procedure, pH was set to 7.4 at room temperature with incubation durations ranging from 10–320 min. This method exhibited several advantages, including increased surface stability and ease of formation, and it was further applied for the detection of AFB1 in alcoholic beverages with a recovery rate of 92 to 102%.

Wei et al. [52] fabricated a new electrochemical aptasensor for the sensitive detection of zearalenone by combining hybridization chain reaction, methylene blue, and exonuclease III for signal amplification and minimizing background current. Exo III operates on double-stranded DNA (dsDNA) and does not require a particular recognition sequence; it may progressively remove single nucleotides along the 3′-5′ direction. In the absence of zearalenone, aptamer, and cDNA coupled to form a double-stranded arrangement on the electrode surface. After the addition of zearalenone, the zearalenone–aptamer complex got detached from the surface of the electrode, allowing the cDNA to start an HCR, which resulted in signal amplification. These reported works on aptamers suggest their immense potential for electrochemical detection.

The advancement in aptamer-based recognition elements for the quantitative analysis of mycotoxins in food samples has facilitated dynamic testing (at room temperature and neutral pH 7.4) of agricultural products, such as maize, wheat, beer samples, and corn samples.

### 2.2. Nanomaterials as Recognition Receptors

Nanomaterials provide structural stability and biocompatibility and offer good electrical properties to the design of biosensors. These provide a wide surface area for effective binding and immobilization of the recognition element of biosensors. High conductivity and excellent catalytic activity of nanostructures also eliminate the requirement of labels and redox mediators during the fabrication of electrochemical sensors. Nowadays, biosensors with different sizes, surface areas, and electrocatalytic characteristics are being developed using nanomaterials [53,54,55]. Numerous nanomaterials and their composites, including carbon nanotubes (CNTs), graphene, gold nanoparticles, silver nanoparticles, and other metal/metal oxide nanocomposites, have been used in the design of sensors because of their excellent optical and electrical properties. These nanocomposites have greatly improved the sensitivity of biosensors by boosting signal production [54]. Carbon-based quantum dots have received great interest recently in various scientific and technical disciplines. These are generally classified into two subgroups, carbon quantum dots and graphene quantum dots [55]. Carbon quantum dots, which have a diameter of less than 10 nm, are fluorescent carbon nanoparticles [56]. Two-dimensional nanocrystals, known as graphene quantum dots, are made up of tiny particles of graphene with lateral dimensions of less than a hundred nanometres [57]. Carbon nanotubes (CNTs) are rolled-up graphenes with the axial properties of the in-plane graphenes translated into them, making them some of the stiffest axial fibers ever made. They are also easily bent, twisted, and buckled, much like graphene [58]. Nanosheets can also serve as labeled aptasensor since they can differentiate between single-stranded DNA and G-quadruplex depending upon their distinct absorption properties [59].

The most widely used nanomaterials for making biosensors are those based on graphene [60,61]. Due to its intriguing characteristics, including its large surface area, higher electrical and thermal conductivity, high stability, biocompatibility, and low cost, graphene is a prospective probe for the electrochemical biosensing of mycotoxins [62]. Additionally, compared to other carbon-based materials, graphene-based probes have superior conductivity and electrocatalytic activity [63]. In the next subsection, the research work reported on different nanomaterials viz. Graphene, CNTs, and metal and metal oxide nanoparticles as recognition receptors in electrochemical sensors have been discussed. 

#### 2.2.1. Graphene in Electrochemical Sensors

Graphene is a 2-dimensional nanosheet of carbon with a higher electrical and thermal conductivity than CNTs, as well as a bigger surface area [64]. Materials based on graphene have been used to make electrodes for devising methods of electrochemical analysis. Electrodes of electrochemical sensors have been coated with graphene to help them recognize various target compounds [65]. In comparison to glassy carbon electrodes (GCE), graphene-coated electrodes can function as effective electrochemical sensors [66]. 

Graphene nanocomposites were successfully coupled with electrochemical devices to increase the electrochemical characteristics of graphene and mycotoxin detection with sensitivity and high selectivity. Phenyl-aminophenyl monolayer (Ph-PhNH_2_/GCE) was used to treat graphene nanosheets before they were covalently connected to electrode surfaces (GNS/Ph-PhNH_2_/GCE) [67]. Utilizing rabbit anti-mouse IgG alkaline phosphatase (RαMIgG-ALP)-AuNPs, a sandwich immunoassay was created with increased sensitivity. Furthermore, using EDC-NHS chemistry, a polyclonal antibody against the clostridium butyricum botulinum neurotoxin type E was immobilized on GNS/Ph-PhNH_2_/GCE surfaces. Within 65 min, the designed immunosensor could successfully detect botulinum neurotoxin type E from orange juice and milk. 

Oxidizing agents, such as NaNO_3_, H_2_SO_4_, and KMnO_4_, have been employed to produce reduced graphene oxide from graphite [68]. Before being put on a glass substrate, the reduced graphene oxide was immobilized onto the surfaces of indium-tin-oxide (ITO). The innovative platform was created by coating anti-AFB1 on the modified glass substrate to enable highly sensitive AFB1 detection. The creation of amide bonds during the binding of the antibody is greatly aided via the treatment of reduced graphene oxide with chemical groups such as−COOH and −OH. A low LOD of 0.12 ng/mL and extreme stability (45 days) were provided by the anti-AFB1/reduced graphene oxide/ITO-based electrochemical sensor. A label-free immunosensor for the quick and accurate detection of AFB1 was developed by the same team using an anti-AFB1/Graphene oxide/indiumtin-oxide immunoelectrode and EDC-NHS reaction chemistry [69]. The modified electrochemical platform showed a number of analytical qualities, such as high sensitivity (639 Ω ng/mL), long-term stability, low LOD (0.2 ng/mL), and broad range (0.5–5.0 ng/mL). This result showed that GO surface modification significantly enhanced the analytical performance of electrodes. The reduced graphene oxide film was similarly deposited on the transparent conductive oxide glass substrate functionalized with bovine serum albumin and anti-AFB1 [70]. 

**Figure 9:**
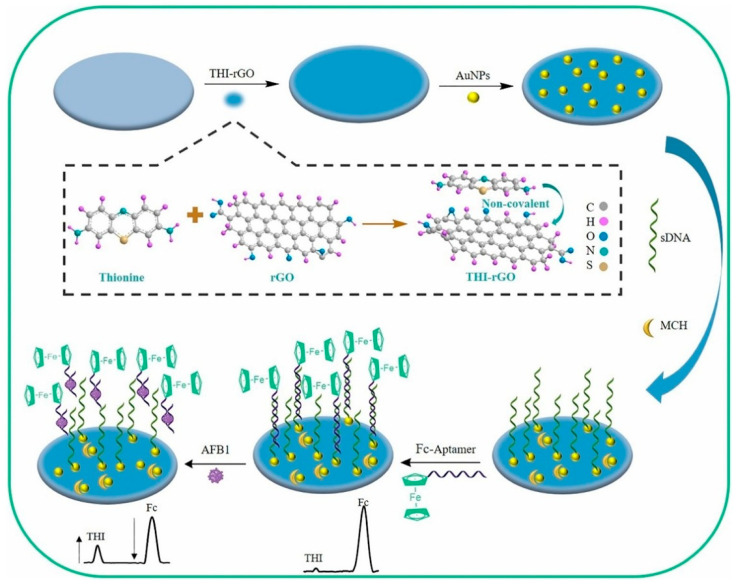
Schematic illustration of the fabrication of the AFB1 aptasensor. Reproduced from [73]. Copyright 2019 Elsevier.

The surface of reduced graphene oxide was coated with platinum nanoparticles to prepare 5,10,15,20-tetraphenyl-21*H*,23*H*-porphine cobalt flat, which was modified with rabbit anti-AFB1 monoclonal antibody to identify AFB1 effectively in bulk samples of peanuts [71]. This method enabled the selective and efficient detection of AFB1 (5.0 pg/mL), which demonstrated the monitoring of AFB1 in samples of food with accuracy and great precision. The same is true for the assembly of pyrrolepropylic acid (PPa) and polypyrrole (PPy) molecules on reduced graphene oxide, which were then used as effective electrochemical devices for the sensing of AFB1 [72]. Through the -COOH group of PPa, the anti-aflatoxin B1 antibody joined covalently to the functionalized reduced graphene oxide, and the presence of PPy improved the detector’s stability and electroactivity stability. The created impedimetric immunosensor was used with great sensitivity and particularity in spiked corn samples to analyze aflatoxin B1. Thionine-derivatized reduced graphene oxide (THI-rGO) has been reported as a reference signal generator to analyze aflatoxin B1. The working electrode was a customized GCE, with Pt wire and an Ag/AgCl electrode serving as the counter electrode and reference electrode, respectively. The ratio of current to intensity was utilized as a signal to detect AFB1 because when AFB1 was present, the Fc-apt-AFB1 couple was released from the electrode surface, causing a drop in impedance and a rise in ITHI (Figure 9) [73].

In the immunoassay for aflatoxin B1, Linting et al. [74] utilized an ionic fluid containing nanocomposites (graphene/conducting polymer/AuNPs). An ionic fluid nanocomposite film was created by combining graphene oxide, 2,5-di-(2-thienyl)-1-pyrrole-1-(*p*-benzoic acid), and gold nanoparticles and electrodeposited on the surface of gold electrodes with nanocomposites. Fast electron transport was considerably accelerated by the graphene and gold nanoparticles. The role of ionic fluid was to connect with the antibodies and create a micro-environment that could improve the efficiency of the electrodes. These distinctive characteristics were crucial in developing a simple immunoassay technique for AFB1 detection, which had 10^−15^ M LOD and more than three months of stability. The sensor had been used successfully for accurate and precise AFB1 detection in food samples. Anti-Ochratoxin A antibody and amine-terminated dendrimer (PAMAM) on graphene oxide nanosheets with manganese ions had been utilized to create a novel impedimetric immunosensor [75]. These authors investigated a cutting-edge idea in EIS for precise OTA identification that was predicated on the potent performance of signal amplification brought on by an efficient immediate activator. By performing a normal immunization between OTA and the immobilized OTA-bovine serum albumin, anti-OTA-graphene oxide nanosheets were first added to the electrode. The oxidation of 4-chloronaphthalen-1-olwas then made possible without the use of H_2_O_2_ by the formation of MnO_2_ when Mn^2+^ ion and KMnO_4_ undergo an in-situ redox process. This innovative tool helped to create an easy electrochemical analytical procedure for the measurement of OTA in actual red wine.

Incorporating metal nanoparticles into graphene oxide nanosheets significantly increased the performance of immunosensors. In order to detect AFB1 without the usage of a label, nickel nanoparticles were coated on reduced graphene oxide nanosheets and employed as an immunosensor [76]. The production of nanocrystalline nanostructures on the reduced graphene oxide–nickel nanoparticle sheets significantly increased the heterogeneous electron transport rate and improved the electrocatalytic characteristics of the material. It was possible to detect AFB1 at a low concentration (0.16 ng/mL) as the probe’s sensitivity was considerably improved (129.6 A ng^−1^ mL cm^2^). An aerogel of reduced graphene oxide decorated with a single DNA strand (single DNA strand/aerogel of reduced graphene oxide) has been added to a spinning disc electrode and utilized as an impediment biosensor for sensitive and specific determination of AFB1 [77]. The impacts of the electro-redox intermediator and the hydraulics effect were researched by authors to enhance the efficiency of the sensor.

Additionally, in a phosphate buffer solution, the cyclic voltammetry response of single DNA strand/aerogel of reduced graphene oxide electrode was examined in the presence of AFB1 at three charges of the redox intermediator, including neutral FeCH_2_OH, cationic Ru(NH_3_)_6_^3+^, and anionic Fe(CN)_6_^4−^. When utilizing the neutral redox mediators FcCH_2_OH (825 mA cm^−1^), it was found that the modified electrode (single DNA strand/aerogel of reduced graphene oxide electrode) displayed stronger action current than both Fe(CN)_6_^4−^ and Ru(NH_3_)_6_^3+^ (615 mA cm^−1^). Due to the cyclopentadienyl ring of the neutral FcCH_2_OH and the aromatic rings of the single DNA strand/aerogel of reduced graphene oxide electrode–electrode interactions, the neutral FcCH_2_OH has significantly interacted with the electrode surface. However, due to attractive or repulsive electrostatic interactions, the electrode surface did not strongly interact with either the cationic (Ru(NH_3_)_6_^3+^) or anionic (Fe(CN)_6_^4−^) mediators. Due to the suppression of charge transfer, a significantly low LOD of 0.04 ng/mL was attained at a higher revolving speed.

For electrochemiluminescent immuno analysis of AFM1in milk, Fe_3_O_4_ nanoparticles were grafted onto the graphene oxide surface to create magnetized graphene nanocomposites [78]. The CdTe quantum dots antibody was used as a signal label in their study, along with magnetic Fe_3_O_4_-graphene oxide as an adsorbent. The signal label (AFM1 Ab1/cadmium telluride-CNT) was then created by decoratingAFM1 antibody onto the surface of the cadmium telluride quantum dots-CNT nanocomposite. Finally, using the sandwich model, an immunocouple was created between Fe-graphene oxide and AFM1 Ab1/cadmium telluride CNT, resulting in the emission of a potent electrochemical signal that could be utilized to detect AFM1. With 0.0003 ng/mL LOD, this approach was employed to monitor AFM1 in dairy products. These studies showed that due to the outstanding qualities of carbon nanomaterials, such as CNTs, graphene, and their derivatives, they are ideal candidates for electrochemical methods for testing mycotoxins. Therefore, using such materials enables the creation of quick and ultrasensitive electrochemical sensors to detect mycotoxin’s actual samples without using sophisticated analytical methods.

#### 2.2.2. CNTs in Electrochemical Sensors

CNTs, particularly multi-walled CNTs and single-walled CNTs, have recently been used as promising candidates for energy devices in analytical and biological sciences due to their outstanding features, such as high conductivity, chemical stability, a high value of surface/volume ratio, high mechanical strength, and biocompatibility, which are the reasons of their performance [79,80,81].

Immobilizing single-walled CNTs with different inorganic and biological compounds has increased the electrical resistance, electron mobility, and biocompatibility of single-walled CNTs for a variety of sensing applications [82]. An electrochemical approach for ultra-level thrombin detection in biofluids was developed by [83]. Chen et al. [66] and Li et al. [84] used single-walled CNTs as electrochemical sensors for the sterigmatocystin and AFB1 detection, respectively, after functionalizing with chitosan and AF-oxidase.

Numerous enzymes were bonded covalently to the MWCNTs’ surfaces because of their enormous surface area, indicating that the MWCNTs serve as supports for the enzymes. Proteins can be covalently conjugated onto carboxylated CNTs using 1-ethyl-3-(3-dimethylaminopropyl)carbodiimide as a cross-linker. It is crucial to distinguish between covalent attachment/ adsorption during the immobilization mechanism since CNTs have a better affinity for a variety of proteins through both electrostatic and hydrophobic interactions [85]. In a similar manner, AF detoxifizyme was mounted on the MWCNTs surfaces and employed for the sensitive sensing of sterigmatocystin [86] and ochratoxin A [87]. Chemical vapor deposition was used to synthesize MWCNTs, and after being exposed to concentrated HNO_3_/H_2_SO_4_, carboxylate groups in significant amounts were produced on their surfaces [88]. The carboxylated MWCNTs were functionalized further with monoclonal anti-aflatoxin B1 which then served as electrodes to detect aflatoxin B1. With great selectivity, the suggested electrochemical sensor provided a lower LOD of 0.25 ng/mL.

MWCNTs were immobilized onto GCE, and then Prussian blue, chitosan, and glutaraldehyde were injected by Fang et al. [89]. The modified electrodes were utilized as a biosensor to detect *Clostridium difficile* toxin B after being treated with an anti-*Clostridium difficile toxin* B antibody. Likewise, single-walled CNTs and microcystin-LR antibodies were added to the paper to enable electrochemical microcystin-LR detection at 0.6 ng/mL in an easy and affordable manner [90]. Zhang et al. [91] modified GCE by adding carboxylic groups to single-walled carbon nanohorn for the microcystin-LR immunoassay. With a 30 pg/mL LOD, the immunosensor provided an optimum analytical platform to measure microcystin-LR. The results were in close agreement with those based on HPLC. For the electrochemical assay of cholera toxin, modification of Nafion film was obtained via MWCNTs [92]. This technique allowed the recognition of cholera toxin even at 1.0 fg/mL by immobilizing cholera toxin antibodies onto a sandwich immunosensor made up of a poly(3,4-ethylenedioxythiophene) film- MWCNTs-Nafion complex.

The electrochemical assay of microcystin-LR was conducted with a new plastic antibody that has been adsorbed onto CNTs and SPCE [93]. In the study for the potentiometric analysis of microcystin-LR, microcystin-YR, and microcystin-RR, a carbon electrode was embellished with molecularly imprinted multi-walled CNTs, which exhibited 1 ng/mL LOD. Yang et al. [94] modified GCE electrodes for the electrochemical immunosensing of FB1 in maize with single-walled CNTs/chitosan. Antibodies, including anti-fumonisin B1 and anti-rabbit immunoglobulin G, were utilized and were attached to an electrode surface via naphthyl phosphate treatment. This approach enabled us to reach a low detection limit (0.002 ng/mL) under optimal conditions, which is much less than 2–4 mg/L (i.e., the regulatory guidelines of European Union Legislation) for fumonisin B1. With this technique, fumonisin B1 in food samples may be measured with good analytical recoveries.

Similar to this, an electrochemical immunosensor was created for quick and easy AFB1detection in food samples [95]. This method involved bonding of AFB1-bovine serum albumin antigen covalently by activating multiwalled CNT and chitosan surface with -COOH group through EDC/NHS chemistry. An 0.1 pg/mL LOD was attained after MWCNT/chitosan electrode modification. This electrochemical device was further used to successfully identify AFB1 in dietary substances, such as soybeans, corn kernels, and palm kernel cake. CNT-based electrochemical devices were used for the successful monitoring of mycotoxins with good selectivity due to their enormous surface area, cylindrical symmetry, the sp^2^ hybridized carbon bonds, and quasi 1D nature. These characteristics significantly aided in the development of materials with enormous potential for real nanoarchitectonic electronic components [96,97]. In the aforementioned CNT-based electrochemical techniques, electrodes were modified with antibodies and certain enzymes that can specifically adhere to the particular mycotoxin. The binding of antibodies and enzymes has been achieved through adsorption, covalent binding, self-assembly, and entrapment into polymers onto the surfaces of CNTs.

#### 2.2.3. Metal and Metal Oxide Nanoparticles in Electrochemical Sensor

**Figure 10:**
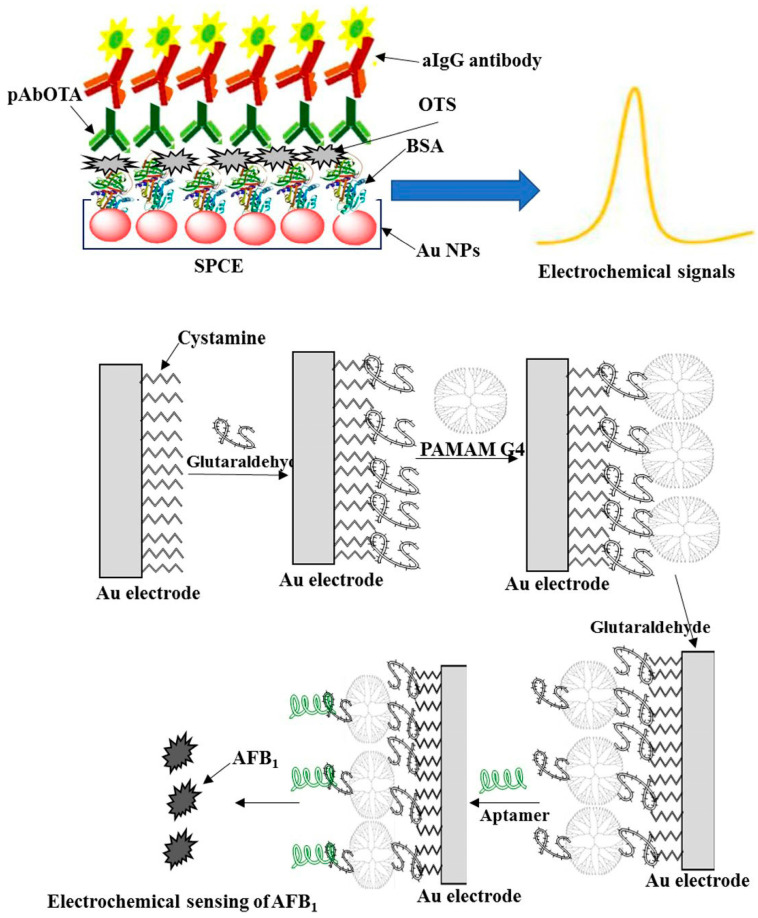
Adsorption of Au NPs, BSA-OTA-pAbOTA, and aIgG antibodies on the surface of SPCE for electrochemical detection of OTA and Schematic for the attachment of aptamer on the surfaces of Au electrode, using cystamine and glutaraldehyde PAMAM G4 as ligands for the formation of self-assembled layer and their interaction with aptamer for specific and selective electrochemical detection of AFB1. Reproduced from [14]. Copyright 2018 Elsevier.

For the electrochemical sensing of different chemicals and biological taxonomic groups in environmental samples, noble metal nanoparticles have been utilized widely as electrodes [98]. In order to build and create user-friendly miniaturized equipment for sensing organic compounds and biomolecules, the combination of noble metals with electrochemical analysis has become a renowned technique. Due to a number of properties, including their ease of fabrication, controllable size and shape, biocompatibility, catalytic and optical properties, chemical stability, powerful adsorption capacity, and electron-transfer kinetics, nanostructured metal oxides have attracted significant attention for sensing applications. In order to quantify mycotoxins in various samples, metal nanoparticles have been widely employed in the design and development of electrochemical sensing probes [11]. One of the essential steps in creating efficient electrochemical sensors is to achieve improved performance via surface modification of electrodes [99]. This led to the physical adsorption of OTA-bovine serum albumin–gold nanoparticles onto the working electrode surfaces, which were then employed as an electrochemical biosensor for the efficient detection of OTA [100]. Bonelet al. [101] developed an immunoassay based on OTA-bovine serum albumin. The manufactured electrodes worked well for OTA electrochemical sensing. The effectiveness of immunosensors for OTA detection has been evaluated using DPV. The outcomes showed that an immunosensor’s ability to perform analysis was considerably impacted by the primary and secondary antibodies’ non-specific adsorption. The incorporation of metal NPs in electrochemical sensing of OTA increased the devices’ analytical capabilities; the produced immunosensor device showed lower LODs than those without nanoparticles. Similar to this, a gold electrode-based cyclic voltammetry technique to detect AFB1 was developed using ferricyanide as a redox couple [102]. This method involved immobilizing AFB1 onto SAM on gold surfaces and using it as an immune capacitive biosensor to detect AFB1 in Brazilian nuts at low LODs of range 7.75×10^−15^ g/mL to 1.35 ×10^−14^ g/mL. A bioimprinting system was later developed by Gutierrez et al. [103] using the protein ovalbumin to create sites with great specificity against aflatoxin B1. A capacitive biosensor with LOD 6×10^−12^ M and great selectivity was created using the bioimprinting technology for the detection of AFB1. A multilayer framework was put together on the Au electrodes using CV and EIS techniques to create an aptamer-based biosensor for the purpose of identifying AFB1 specifically. In their research, poly(amidoamine) dendrimers of the fourth-generation amine-terminated dendrimer (PAMAM G4) were employed to decorate the cystamine-Au electrode and served as a redox mediator for K[Fe(CN)_6_]^−3^/^−4^ [34]. Then, to serve as particular recognizing components for AFB1 detection, modified DNA aptamers were applied to the modified Au NP electrode. AFB1 in food products was successfully detected using the designed electrochemical device. The concept of coating of electrode surface with particular materials (antibodies, proteins, polymers, aptamer, etc.) for the discriminative electrochemical detection of OTA and AFB_1_ is shown in Figure 10 [14].

The food chain is the mechanism by which marine poisons produced by tiny algae accumulate. They have a well-established history of putting humans at risk for a number of neurological and gastrointestinal disorders [104]. For the simultaneous determination of dinophysistoxin-1 and brevetoxin B in seafood samples, a new extremely fast and sensitive complex immunoassay-based electrochemical procedure was formulated [30]. The surfaces of magnetic beads were co-immobilized with monoclonal mouse anti-brevetoxin B and anti-dinophysistoxin-1 antibodies in this study. The created electrochemical sensor was used for both brevetoxin B and dinophysistoxin-1 monitoring at the same time with a broad linear range of 0.005 ng/mL to 0.05 ng/mL. An electrochemical method was created, in which functionalized gold nanoparticles were used with amine-terminated poly(amidoamine) dendrimers for quick detection of brevetoxin B in food products [105]. These scientists reported that amine-terminated poly(amidoamine) dendrimers boosted the conductivity of poly(amidoamine) dendrimers while 3-dimensional poly(amidoamine) dendrimers significantly expanded the electrode’s surface area to capture target analytes with extreme precision. The created sensor made it possible to detect brevetoxin B in food samples at low concentrations (0.01 ng/mL).

**Figure 11:**
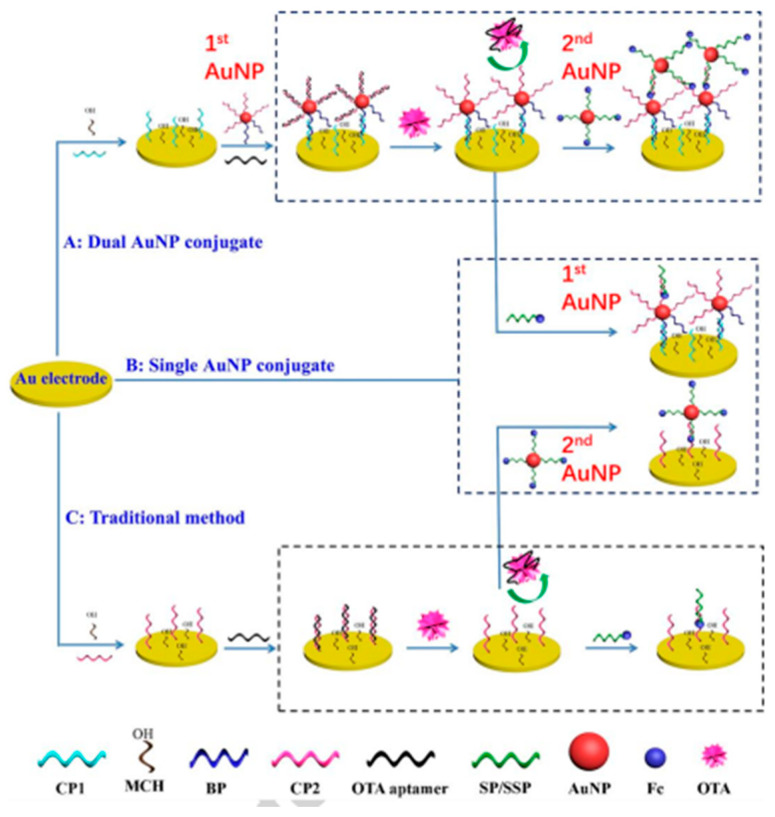
Schematic diagram of the (**A**) Dual AuNP conjugates, (**B**) Single AuNP conjugates, and (**C**) Traditional method (non-AuNPs)-based electrochemical aptasensors for amplified assay of OTA. Reproduced from [41]. Copyright 2018 Elsevier.

For the highly sensitive analysis of OTA, a signal-on aptamer-based electrochemical sensor having a DNA-controlled layer-by-layer assembly of dual AuNP couples was created (Figure 11). Here, five distinct single-stranded DNA strands were used to control the formation of the first and second AuNP conjugates, which exhibited varied oligonucleotide modifications but identical AuNP sizes. Both AuNP conjugates were capable of enhancing the electroconductivity and load abundance of ferrocene (Fc). The identification of OTA with aptamer caused aptamer to be removed from the electrode surface and the first AuNP conjugate, freeing the CP2 probe in the single-strand structure. As a result, there was a direct proportionality between the final signal generated and OTA concentration, and it was anticipated that this novel approach would make an excellent all-encompassing sensing platform for trace-level biochemical analysis of OTA [41].

Thus, the incorporation of nanocomposites and nanostructures in the recognition receptors of electrochemical biosensors has made possible the extremely sensitive sensing of mycotoxins in food samples. The advancement of cutting-edge techniques for creating and modifying nanocomposites and nanostructures with receptors and heteroatoms has considerably enhanced the performance of biosensors. Nanomaterials have contributed to an enhancement in electrode analytical capability for the detection of mycotoxins in comparison to other pure electrode forms. 

### 2.3. Polymer-Based Materials as Recognition Receptors

Developing electrochemical biosensors with an interfacial design derived from polymer-based materials has gained much attention because of its fast and selective sensing response and applicability on a small sample. Electrochemical biosensing through polymeric materials provides high selectivity, high sensitivity, and robustness at a low cost. Polymers having functional structures, such as polypeptides, lipid bilayers, and poly (ethylene glycol) chains, have gained excellent attention due to their continuous use in drug delivery, tissue engineering, and nanoscale self-assembly [106,107]. All these forms of polymers with their specific characteristics are linked with the recognition properties, such as biodegradability, biocompatibility, mimicking catalytic activity, mycotoxins sensing, and antibody-antigen interaction. Materials, such as polypeptides, exhibit different properties when these are attached to polymeric materials, such as methoxy poly (ethylene glycol) [108]. There are various approaches, such as colorimetric immunoassay, electrochemical biosensing, paper-based lateral flow, and fluorescence-based optical sensing, etc., reported for sensing mycotoxins. In this area, many researchers have developed different methods for electrochemical biosensing using polymeric materials. Tan et al. [107] synthesized polymer–enzyme-multiwalled carbon nanotubes (MWCNTs), which showed excellent potential for electrochemical biosensing of mycotoxins and biofuel applications.

In the area of electrochemical biosensing of mycotoxins, Xing et al. [109] designed a material from a green enzyme-linked immunosorbent assay (ELISA) grounded over a single-stranded binding protein (SSB), which was dispersed on a white polystyrene plate and employed for the detection of mycotoxins ZEN, AFB1, OTA in corn samples. 

It showed that when there was a target analyte present, the aptamer−biotin could not bind to the SSB in the presence of mycotoxins, which resulted in the weak yellow color of the solution. A strong interaction between the streptavidin (SA) and biotin, after the addition of substrate/chromogen solution, resulted in a bright yellow-colored solution. From all the obtained result author found the LOD values 112 ng/L, 319 ng/L, and 377 ng/L for AFB1, OTA, and ZEN, respectively.

**Figure 12:**
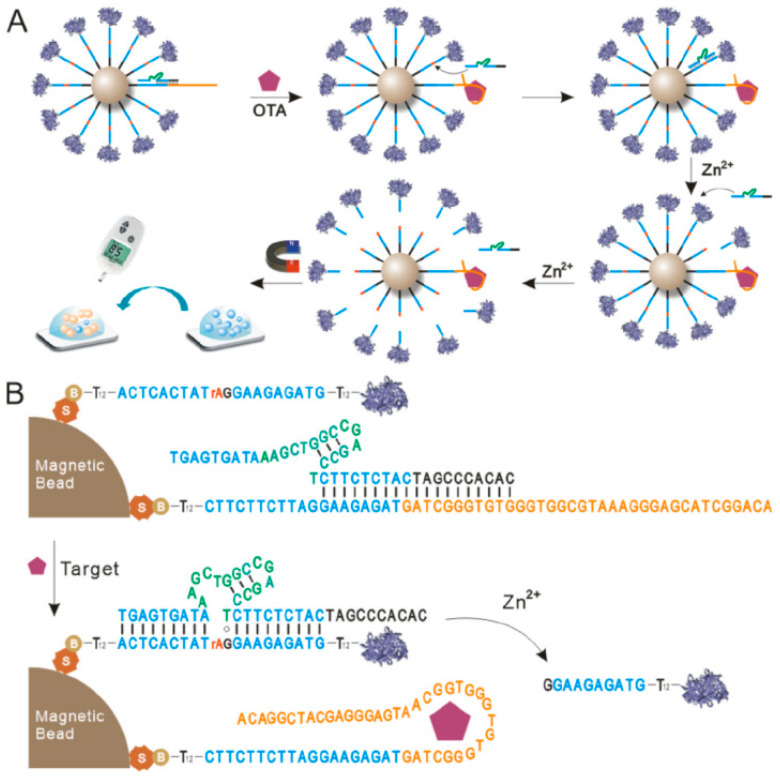
Schematic diagram of apta-sensing platform for OTA detection through DNAzyme amplification. (**A**) stepwise synthesis of magnetic removal beads (MB) and (**B**) OTA brought the displacement of DNAzyme, substrate−DNAzyme complex, and further invertase-labeled substate was released. Reproduced from [110]. Copyright 2021 ACS Publications.

Zhang et al. [110] fabricated a DNAzyme aptamer platform for the determination of OTA using a glucose meter. The magnetic bead-based receptor was used for the recognition of OTA present in food samples. Detection of mycotoxins through magnetic beads was based on the mechanism (Figure 12) where the substrate strand had other 12 T bases at 5′ and 3′ ends as per spacers, and during the whole process of detection, key aptamer contained a total of four compartments. 

During the analysis of mycotoxins, the aptamer arrangement of OTA was attached to the 3′ end instead of the 5′ end; this was probably due to the aptamer, which was situated at the exterior of the spherical nucleic acid, which was more appropriate for the direction of OTA toward the aptamer. The aptamer probe underwent a conformational change in the presence of OTA molecules, which aided in its detection of the lowest limit of 0.88 pg/mL.

**Figure 13:**
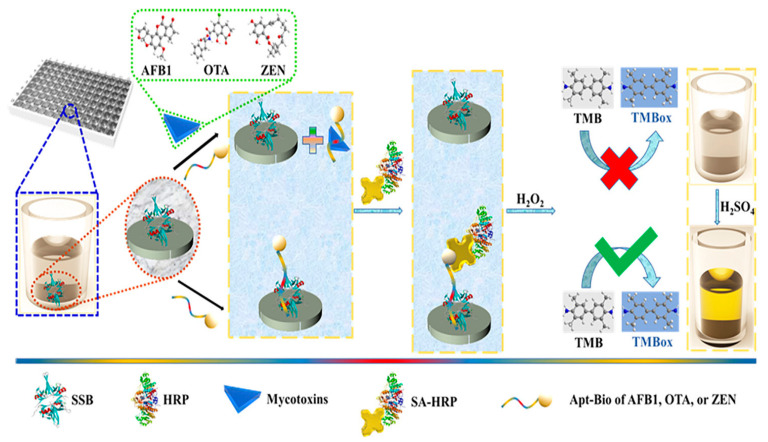
Schematic diagram for synthesis of green ELISA based on SSB-assisted aptamer and step-by-step detection of mycotoxins using the ELISA-based SSB-assisted aptamer. It shows that when there were target mycotoxins present, the aptamer−biotin could not bind to the SSB in presence of mycotoxins, which resulted in weak yellow color of solution. Reproduced from [111]. Copyright 2020 ACS Publications.

Hu et al. [111] developed a material from poly(o-phenylenediamine) that was further used for the analysis of AFB1 (Figure 13). The authors developed the software Zsimpwin for the simulation of EIS results and obtained software without staphylococcal protein A (SpA), which displayed that the DRbrf values exhibited a linear relationship to concentration from 3.0–100 ng/mL with a detection limit of 1.5 ng/mL, which signified that developed impedimetric immunosensor was appropriate for AFB1 detection. Li et al. [112] also developed an economical and portable electrochemical sensing device that was highly sensitive and selective for rapid analysis of AFB1 in rice samples. The immunosensor exhibited a LOD of 5 ng/mL in one hour, including the incubation time.

**Scheme 1:**
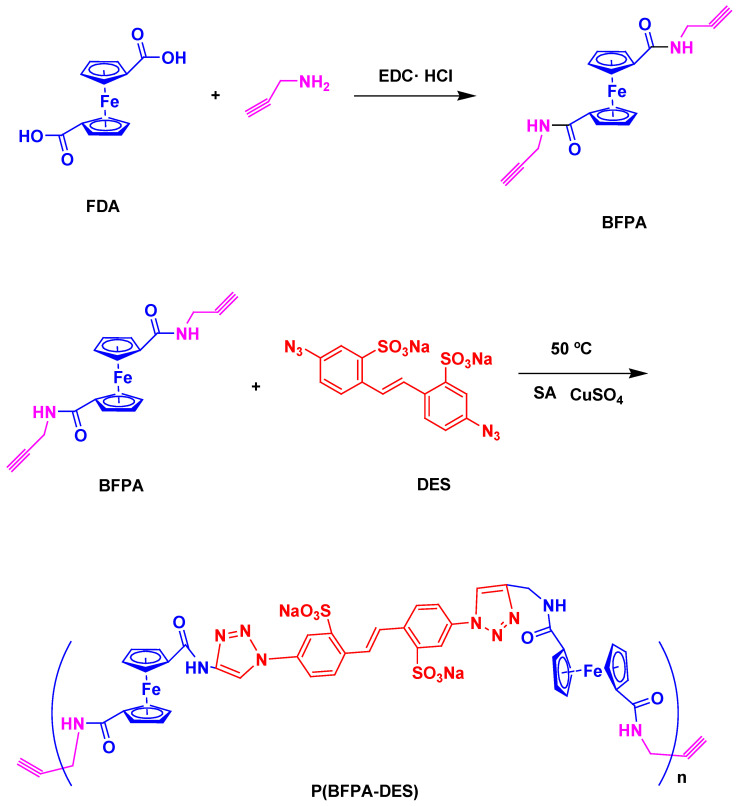
Synthesis of P(BFPA-DES).

Liu et al. [113] developed a simple and cost-effective electrochemical sensing platform approach through the click polymerization technique for the determination of AFB1. For this sensing approach, bis-ferrocenoyl propargylamide (BFPA) was synthesized using the chemical method. Thereafter, P(BFPA-DES) was prepared by click polymerization of BFPA and disodium salt tetrahydrate (DES) (Scheme 1). The azide group, which was attached at the end of c-DNA, covalently formed a bond with attained electroactive polymer P(BFPA-DES). For AFB1, aptamer acted as a capture probe, which further self-assembled over the surface of the Au electrode through Au–S bonding. When azide groups were modified, the complementary DNA (c-DNA) was hybridized with the aptamer, and click chemistry was responsible for the inclusion of polymer. The obtained electrochemical aptamer sensor exhibited great selectivity, sensitivity, and reliability for analysis of AFB1 in real samples with potential applications in the area of food safety and hygiene.

**Scheme 2:**
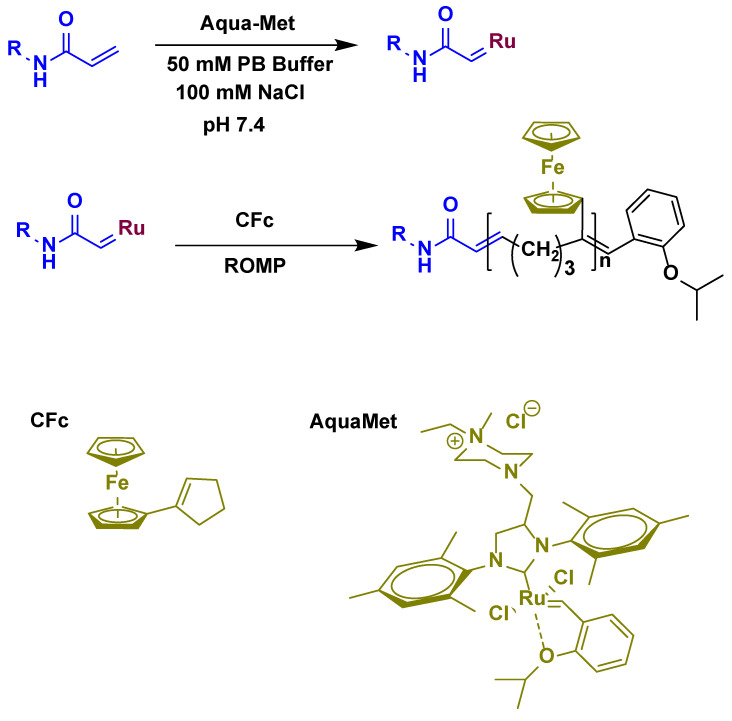
Reaction mechanism of ring-opening polymerization (ROMP).

For the detection of AFB1, Yang et al. [114] also fabricated an electrochemical sensor via ring-opening metathesis polymerization (ROMP) (Scheme 2). For the synthesis of the sensor, the Au electrode was immobilized with aptamer and AFB1 specific antibody, followed by grafting of cyclopentenyl ferrocene units using AquaMet catalyst, which resulted in the formation of long chain polymeric electroactive structure. This developed polymeric electro-sensor exhibiting a LOD of 0.34 fg/mL with a linear detection range of 2 fg/mL to 2 ng/mL. The prepared sensor was highly specific and selective because of the excellent interaction of the target with aptamer and antibody. In addition, the developed sensor exhibited an excellent tendency for quantification of AFB1 in real samples with 92.91% to 107.20% recovery. 

Joshi et al. [115] proposed work in this area to develop a double 3-plex benchmark approach for the analysis of mycotoxins present in barley. In this regard, the author prepared surface plasma resonance (SPR) with flat Au biosensor chips, which were effective in the determination of six mycotoxins present in barley. 

For the sensing of OTA, Gaun et al. [116] fabricated a novel method based on tetrahedral DNA nanostructure (TDN) combined with cobalt metal–organic framework (Co-MOF), with high reproducibility, high stability, high sensitivity, and good versatility. The developed sensor had excellent selectivity to quantify OTA in red wine with a limit of detection of 0.3 fg/mL.

A sensor for conductometric analysis of OTA in olive oil samples has been proposed by Dridi et al. [117]. This electrochemical sensor was derived through the immobilization of thermolysin (TLN) into a polyethylene (PEI)/polyvinyl alcohol (PVA) matrix, which crosslinked with glutaraldehyde and contained gold nanoparticles (AuNPs). The developed sensor displayed a sensitivity of 597 SM^−1^ at a working pH of 7 and a temperature of 298K. Thus, PEI/PVA hydrogel created an excellent aqueous environment for the enzyme. Moreover, the bonding between the protonated amino group present on PEI and the negative charge of thermolysin and citrated AuNPs also helped their dispersion over the polymer blend. When AuNPs were not dispersed over the polymer blend, no conductometric signal was observed, which indicated the insulating property of crosslinked PEI/PVA hydrogel films. The author observed that the developed sensor had a LOD of 1 nm for OTA.

In this area, Wang et al. [118] designed a molecularly imprinted polymer (MIP) for the selective capture of AFB1 from food samples. MIPs come under the category of biomimetically synthesized material through the bulk polymerization method, which was used for the recognition of target molecules based on cavities created on the template surface. Here, the authors synthesized a 3D microporous magnetic inverse photonic crystal microsphere (MPCM) as a supporting material. In the prepared material, MPCM@MIP 5,7-dimethoxycoumarin acted as a template, and methacrylic acid monomer worked selectively toward AFB1. MPCM@MIP displayed a detection limit of 0.4 ng/mL.

In the field of molecularly imprinted polymer, Hu et al. [119] prepared an effective ratiometric sensor for the detection of ochratoxin A. In these aspects, MIP was prepared from poly ionic-liquid, gold nanoparticles, and flavin mononucleotide-decorated carbon nanotubes-MoS_2_nanosheets couple. A MIP-based electrode was used for the detection of OTA in real beverage samples. The samples were tested with HPLC for comparison. Under optimized conditions of the MIP electrode, the LOD was 14 nM, and the linear range varied from 0.5–15 μM.

Pacheco et al. [120] also worked on the electrochemical sensing of OTA in bear and wine samples, using GCE, modified with MIP and multi-walled CNTs. In this work, MIP polymeric films were fabricated by electro-polymerization of pyrrole in the presence of template OTA, and a prepared sensor was used to investigate the electrochemical oxidation of OTA via DPV and CV. The obtained results showed a direct correlation between OTA concentration and current intensity. Thus, the author was able to prepare the sensitive electrochemical sensor with reproducibility for the detection of OTA in the range of 0.050–1.0 M, and the detection limit was calculated as 0.0041 M to 0.014 M. Another effective electrochemical biosensing of AFB1 was proposed by Akgonullu et al. [121], using MIP with Au nanoparticles coated over the SPR gold chip surface. Sensing of AFB1 was based on the pre-complexed formation between N-methyl-L-phenylalanine and AFB1 as a template molecule and respectively used functional monomer. The electrochemical detection of AFB1 in the ground corn sample displayed a LOD of 1.04 pg/mL, and the linear detection range was varied from 0.1pg/mL to 10.0 ng/mL.

Similarly, Munawar et al. [122] designed MIP nanoparticles for the electrochemical biosensing of FB_1_, which is a carcinogenic mycotoxin found in animal feed and food items. A conducting polypyrrole-(zinc porphyrin) composite was used to fabricate the MIP nanoparticles over the Pt electrode via the electro-polymerization method. The developed chemosensor was capable of detection of FB_1_with high sensitivity from 1 fM–10 pM with LOD 0.03 fM to 0.7 fM. A molecularly imprinted electrochemical sensor based on metal–organic framework was designed by Jiang et al. [123] for the detection of AFB_1_. The sensor was developed by electro-polymerization of p-amino thiophenol, which was further functionalized with gold nanoparticles. The cavities formed after extraction of the template were able to recognize the AFB_1_ specifically via π-π interaction with aniline moieties. The prepared electrode was used for the detection of AFB1 in spiked rice sample with LOD 3 fM, and the linear range varied from 3.2 fM to 3.2 μM. Another effective electrochemical sensing technique for AFB1 and FuB1 was proposed by Singh et al. [124]. MIP matrix was synthesized from aniline via the chemical oxidative polymerization method. The author used CV and DPV for electrochemical sensing of AFB1 and FuB1 with a linear range of 1 pg/mL to 500 ng/mL, and detection limits were 0.313 pg/mL and 0.322 pg/mL for AFB1 and FuB1, respectively. Based on the polymerization of o-phenylenediamine and their fabrication over the screen-printed gold electrode, Radi et al. [125] also proposed a strategy for analysis of zearalenone using EIS and CV with LOD 2.5 ng/mL and determination varied up to 200 ng/mL.

## 3. Conclusions and Future Prospects

Electrochemical biosensing has proven as an effective tool for the detection of mycotoxins. In this review, different types of recognition receptors used for the detection of mycotoxins have been elaborated. The unique emerging materials include CNTs, molecular imprinting polymer, graphene, aptamer, nanomaterials, DNAzymes, antibodies, and peptides. They have paved the way for electrochemical sensing with huge potential for the facile detection of mycotoxins. Few biosensors have been commercialized for the detection of mycotoxins in real food samples. There is a need for more research to check their efficacy under real-life conditions. The shelf life and reusability of sensors are two main aspects that need to be explored further. Reversible binding and regeneration of recognition receptors after use can increase the shelf life and cost-effectiveness of electrochemical sensors. Another challenge that needs to be addressed is the miniaturization of the electrode of the sensor and the analyte sample containing mycotoxins. On-site detection of mycotoxins using portable, handy, and easy-to-use sensors is another important aspect that needs to be explored in order the cut the cost of sophisticated instrumentation and skilled manpower. Research on the monitoring of spoilage of food due to mycotoxins contamination at the industrial level using smartphone devices is the need of the hour so that the spoilage is automatically detected and huge economic loss to the industry can be avoided. It can be concluded that electrochemical biosensing exhibit excellent sensitivity, a wide range of detection, easy handling, real-time measurement, and a lower limit of detection.

**Table 1:** Electrochemical biosensing of mycotoxins using recognition receptors.

Analyte	Electrode Materials	LOD (ng/mL)	Linear Range (ng/mL)	Analytical Method	Real Samples	Ref.
AFM1	Fe_3_O_4_-GO- CdTe QDs	0.0003 *	0.001–0.001 × 10^5^ *	Multifuncional electrochemical analytical system	milk	[78]
AFB1	Apt/NR-TCA/GCE	0.015	0.03–30	DPV	Beer and wine	[22]
	Apt-CSs-modified	0.002 *	0.007–0.5	DPV	Human serum, Grape juice	[50]
	SPE	0.6 × 10^−7^ *	0.1 × 10^−9^ to 1 × 10^−4^	SWV	Corn	[51]
	Screen-printed interdigitated microelectrode (SPIMs)	10	5 to 20	EIS and CV	Rice	[112]
	MPCM@MIP	0.4	5 to 1000	UV and HPLC Analysis	Soysauce and vinegar	[117]
	Thiol-modified aptamer	0.000034 *	0.000002 to 2 *	SWV, EIC, and CV	Medicine	[114]
	Au NPs	0.93 × 10^−6^ *	0.93 × 10^−6^ to 0.9 *	CV	Rice	[123]
	MIP/Au NPs	0.001 *	0.0001 to 10.0	SPR	Ground corn	[121]
	Multi-walled CNTs-Pt	0.46 *	0.9–207.95 *	Chronamperomety	Corn, rice, and wheat	[84]
	MWCNTs- COOHITO	0.08	0.25– 1.375	CV	-	[88]
	MWCNTs-CS	0.0001 *	0.0001– 10	DPV	Palm Kernel Cake and Feed Samples	[95]
	rGO-ITO	0.12	0.125–1.5	CV	-	[68]
	GO-Au NPs	0.23	0.5–5	CV	-	[69]
	rGO-transparent conductive oxidase	10^−10^ *	10^−10^–10^−6^ *	CV	Corn	[70]
	PtNPs-CoTPP- rGO	0.0015 *	0.005–5.0	DPV	Peanut	[71]
	rGO-PPy-PPa	0.000001 *	0.000001–0.01 *	EIS	Corn	[72]
	G-polymer-Au NPs	0.3 × 10^−6^ *	0.3 × 10^−6^– 0.910^−3^ *	CV	Peanut, rice, milk flour, and soyabean	[74]
	rGO–Ni NPs- ITO	0.16	1.0–8.0	CV	-	[76]
	Gold electrode-imprinted Protein	1.97×10^−3^ *	1–1000	CV	Nut	[103]
	Gold–PAMAM dendrimers	0.124 ± 0.03 *	0.1–10 nM	CV and EIS	Rice, corn	[34]
AFB1 and FuB1	Polyaniline	0.0003 and 0.00032 *	0.001 ng/mLto 500 ng/mL *	CV and DPV	Rice, barley, peanuts, oat	[125]
FB1	Pt electrode	0.5 × 10^−5^ *	0.031 to 3.12 *	DPV and EIS	Maize Sample	[122]
	SWCNTs-CS	0.002 ^*^	0.01–100	EIS	Corn	[94]
OTA	dsDNA/MCH/ssDNA modified AuE	0.001 *	0.005–10.0	DPV	Mildewed wheat starch sample	[48]
	Fc and βCD	0.0004 *	0.001 to 10 *	CV	Peanut oil	[108]
	Au NPs@Co-MOF	0.000031 *	0.000001 to 0.00005 *	EIS, CV, and DPV	Red wine	[116]
	Au NPs embedded PEI/PVA hydrogel	0.4 *	Upto 24.2 *	TLN Conductometric biosensor	Olive oil	[118]
	MIP/Au NPs/PIL-FMNS/CNT-MoS2/GCE	5.6 *	2 × 10^5^–6 × 10^5^ *	EIS and CV	Beer, red wine, and Chinese liquor	[119]
	MWCNTs/MIP	1.7 *	20.19–403.81 ng/mL *	CV and DPV	Beer and Wine	[120]
	CNT-ITO	0.0025 *	0.0025–0.06 *	DPV	Serum	[87]
	Au NPs-BSA- OTA	0.86	0.3–8.5	DPV	Wheat	[101]
	GO-PAMAM- Mn^2+^	0.0005 *	0.0001–30 *	EIS	Red wines	[75]
ZEA	AuE	0.00013 *	5.0 × 10^5^–50 *	DPV	Corn sample and beer	[52]
	MIP/Au NPs	2.5 *	Up to 200 *	EIS and CV	Corn	[125]

* The units have been made uniform for comparison purposes. Electrochemical Impedance Spectroscopy (EIS), Linear-Sweep Voltammetry (LSV), Cyclic Voltammetry (CV), Differential-Pulse Voltammetry (DPV), Square-Wave Voltammetry (SWV), High-Performance Liquid Chromatography (HPLC), Ultraviolet (UV), Thermolysin (TLN), Surface Plasmon Resonance (SPR), Zearalenone (ZEA), Ochratoxin A (OTA), Fumonisin B1 (FuB1), Fumonisin B1 (FB1), Aflatoxin B1 (AFB1), Aflatoxin M1 (AFM1), and details of electrode material in text.

## Data Availability

Not applicable.
